# New records of fish parasitic isopods of the gill-attaching genus *Mothocya* Costa, in Hope, 1851 from the Virgin Islands, Caribbean, with description of a new species

**DOI:** 10.3897/zookeys.439.8093

**Published:** 2014-09-10

**Authors:** Kerry A. Hadfield, Paul C. Sikkel, Nico J. Smit

**Affiliations:** 1 Water Research Group (Ecology), Unit for Environmental Sciences and Management, Potchefstroom Campus, North West University, Private Bag X6001, Potchefstroom, 2520, South Africa; 2 Department of Biological Sciences, Arkansas State University, P.O. Box 599, State University, AR, 72467, USA

**Keywords:** Cymothoidae, *Mothocya*, gill chamber, fish parasite, Caribbean Sea, St. Thomas, St. John, Guana Island

## Abstract

Two species of *Mothocya* Costa, in Hope, 1851 are reported from the Virgin Islands. *Mothocya xenobranchia* Bruce, 1986 was collected from St. John Island from the gills of the Atlantic needlefish, *Strongylura marina*, which is a new locality record and also confirms a previously uncertain host identity. *Mothocya bertlucy* **sp. n.** is described from St. Thomas, St John and Guana Islands, from the gills of the redlip blenny, *Ophioblennius macclurei*, the first record of a blenny as host for any *Mothocya*. The distinguishing characters of *Mothocya bertlucy* **sp. n.** include its small size (< 9 mm) and eyes, the slender pleotelson with a narrowly rounded caudomedial point, extended uropod peduncle and uropods which do not extend past the pleotelson posterior margin, and the narrow pleon which is only slightly overlapped by pereonite 7.

## Introduction

Cymothoid isopods are one of the most recognisable groups of isopods to fisherman and anglers (Smit et al. 2014). These large (> 6 mm) aquatic parasites are commonly found on the external surface, inside the buccal cavity, or in the branchial cavity of their fish host. Cymothoids removed from the gills are often asymmetrical in body shape, twisted slightly due to the shape of the gill arches and operculum in the branchial cavity (Kensley and Schotte 1989).

In some cases, these parasites cause gill and branchial filament damage (Kroger and Guthrie 1972, Colorni et al. 1997). Williams and Williams (1978) commented on the discolouration and considerable erosion of the gill filaments and opercular flap in some fish they studied. Rokicki (1982) noted atrophy of the gill filaments which automatically affects the fish’s development; and Colorni et al. (1997) reported on deformed and calcified gill rakers as well as gill filaments which were dystrophic and fused together with total obliteration of both primary and secondary lamellae.

One of these gill-attaching cymothoid genera is *Mothocya* Costa, in Hope, 1851. Historically the systematics and biology of this genus had not been considered problematic, but Bruce (1986) showed that *Irona* Schioedte & Meinert, 1884 and *Mothocya* were synonymous and that many of the species were misidentified, which had led to the misrepresentation of their hosts and distributions. Bruce (1986) comprehensively reviewed *Mothocya* and corrected many of these errors, revising seven species and describing 18 new species. Since then, another four species have been described (WoRMS 2014), making a total of 29 valid *Mothocya* species in the world (Smit et al. 2014).

There are six known species of *Mothocya* in the Caribbean Sea. These are *Mothocya argenosa* Bruce, 1986 (Bermuda; Florida and Georgia, USA; Cuba; and the British Virgin Islands); *Mothocya bermudensis* Bruce, 1986 (Bermuda; Haiti; Saint Barthélemy, Leeward Islands); *Mothocya bohlkeorum* Williams & Williams, 1982 (Florida, USA; Bahamas; Saint Eustatius, Leeward Islands; and Puerto Rico); *Mothocya nana* (Schioedte & Meinert, 1884) (Florida, Georgia and Maryland USA; Saint Barthélemy, Leeward Islands; and Panama), *Mothocya omidaptria* Bruce, 1986 (Brazil and West Indies), and *Mothocya xenobranchia* Bruce, 1986 (Florida, USA; and Venezuela). To date there are no known species recorded from the US Virgin Islands and only one species known from the British Virgin Islands (*Mothocya argenosa*). The new species described here increases the number of species known from the Caribbean to seven.

## Methods

Collections were made from the Virgin Islands, specifically St. John, and St. Thomas, US Virgin Islands, and Guana Island, British Virgin Islands, in the Caribbean Sea during 2013 as part of a study on blood parasites of Caribbean reef fishes. Atlantic needlefish (*Strongylura marina*) were collected near the surface at night by snorkelers using hand nets, while redlip blennies (*Ophioblennius macclurei*) were collected by hand nets during the day from reef habitat in shallow bays by snorkelers or divers. Isopods were removed from the gills of their infected hosts using forceps, preserved in 70% ethanol, and processed according to techniques described in Hadfield et al. (2010, 2011). Species descriptions were prepared in DELTA (Descriptive Language for Taxonomy, see Coleman et al. 2010) using a general Cymothoidae character set (as in Hadfield et al. 2013, 2014). Ratios and measurements were rounded off to one decimal place and were made using maximum values of the specific measured article. Classification follows Brandt and Poore (2003). Host nomenclature and distribution are from FishBase (Froese and Pauly 2014).

**Abbreviations.** AMNH – American Museum of Natural History, New York, NY, USA; TL – total length; USNM – National Museum of Natural History, Smithsonian Institution, Washington, DC, USA; W – width.

## Taxonomy

### Family Cymothoidae Leach, 1814

#### Genus Mothocya

Taxon classificationAnimaliaIsopodaCymothoidae

Costa, in Hope, 1851

Mothocya Costa, in Hope, 1851: 48. – Trilles 1968: 168. – Monod 1971: 174. – Bruce 1986: 1092–1095. – Trilles 1994: 197.
Irona Schioedte & Meinert, 1884: 381. – Stebbing 1905: 27. – Richardson 1905: 265. – Hale 1926: 218. – Monod 1971: 174. – Kussakin 1979: 307. – Trilles 1994: 166.

##### Diagnosis.

Body not vaulted, widest at pereonite 5, usually twisted to one side. Cephalon with rostrum folded back, anterior margin rounded. Antennae widely separated, antennula longer and more stout than antenna. Eyes distinct. Maxilliped article 3 with 3–5 recurved robust setae; without oostegite lobe. Maxilla mesial lobe partly fused to lateral lobe. Maxillula simple. Pereonite 1 anterolateral angles slightly extended around cephalon. Pleon subequal to pereon. Pleonite 1 partly concealed by pereonite 7. Coxae 5–7 dorsally visible, projecting posteriorly past respective somite; large, and rounded, reniform. Brood pouch formed from coxae 2–4 and 6. Pereopods without carina, never enlarged or with protrusions. Pleopods simple, without setae. Pleopods 3–5 with lamellar proximomedial lobe, frequently with peduncle lobe. Uropod peduncle without retinaculae, exopod longer than endopod.

##### Type species.

*Mothocya epimerica* Costa, in Hope, 1851; by subsequent designation (Bruce 1986). Costa, in Hope (1851) described three species, *Mothocya contracta* Costa, in Hope, 1851, *Mothocya detecta* Costa, in Hope, 1851 and *Mothocya epimerica* of which only *Mothocya epimerica* is recognised as a valid species.

##### Remarks.

Female *Mothocya* are often twisted to one side due to the confines of the gill chamber. *Mothocya* can be identified by the asymmetrical body shape, antennula longer than the antenna, a maxilliped with an oostegite lobe and the brood pouch from coxae 2–4 and 6. Males are smaller and not twisted, with appendix masculina on pleopod 2.

A detailed diagnosis of *Mothocya* was given by Bruce (1986), including female and male characters as well as additional characters for the genus. The current diagnosis is a shortened and updated version with more information on the main defining characters such as the body, pleopod and uropod morphology. These important characteristics are very useful in species identifications, as is the host species with some *Mothocya* species being host species or host genus specific.

Bruce (1986) synonymised *Irona* with *Mothocya*, with many of the *Irona* species actually being junior synonyms for *Mothocya* species. The validity of the genus *Irona* was considered uncertain for many years (Monod 1923, 1971, Trilles 1968) after Schioedte and Meinert (1884) described it as well as redescribing *Mothocya* in the same paper. Bruce (1986) described 18 new species of *Mothocya* in his review, nine of which had synonymies from earlier misidentifications. Many species appear very similar in general appearance, with the antennulae, antennae, mouthparts and pereopods uniform across species and thus not very informative at species level (Bruce 1986).

When looking at individual characters, *Mothocya* can be distinguished from other gill-inhabiting genera. *Elthusa* Schioedte & Meinert, 1884 is similar to *Mothocya* and can be distinguished by the antennula being shorter than the antenna (longer in *Mothocya*), maxilliped article 3 is slender with setae (robust and without setae in *Mothocya*), and the pereopod dactyli are relatively short whereas they are long and robust in *Mothocya* (Bruce 1990). *Ichthyoxenus* Herklots, 1870, differs from *Mothocya* with the antennula being shorter than the antenna, having a strongly ovate and vaulted body, as well as a narrow pleon and short and rounded coxae.

*Mothocya* occurs in all oceans and is predominantly tropical and subtropical in its distribution. Currently 29 species names are valid (*Mothocya contracta* Costa, in Hope, 1851 designated as *nomen dubium*), with four species described since Bruce’s (1986) monograph.

#### Mothocya xenobranchia

Taxon classificationAnimaliaIsopodaCymothoidae

Bruce, 1986

Figs 1 –2

Mothocya xenobranchia Bruce, 1986: 1116–1119, figs. 13–14. – Trilles 1994: 203. – Bunkley-Williams et al. 1998: 29. – Bunkley-Williams et al. 2006: 178. – Schotte et al. 2009: 983.

##### Material examined.

♀ (15.0 mm TL; 10.0 mm W), ♂ (9.0 mm TL; 4.0 mm W) collected from Lameshur Bay, 18°18'59"N, 64°43'25"W, St. John Island, US Virgin Islands, from the gills of the Atlantic needlefish (34 mm TL), *Strongylura marina*, 18 May 2013, coll. Nico J. Smit (AMNH_IZC 00197448).

###### Ovigerous female.

Body moderately twisted, 1.4 times as long as greatest width, strongly arched longitudinally, widest at pereonite 3, most narrow at pereonite 1, lateral margins slightly convex. Cephalon 0.7 times longer than wide, visible from dorsal view, subtriangular. Eyes oval with distinct margins, 0.2 times width of cephalon, 0.4 times length of cephalon. Coxae 2–3 narrow; 4–7 large, rounded and produced, slightly produced past pereonite margin. Pereonites 1–4 increasing in length and width; 5–7 decreasing in length and width; becoming more progressively rounded posteriorly. Pleon with pleonite 1 largely concealed by pereonite 7; pleonites posterior margin smooth, mostly concave; pleonites 2–5 partially overlapped by pereonite 7; pleonite 5 posterior margin straight. Pleotelson 0.6 times as long as anterior width, dorsal surface smooth, anterolateral margin recessed, lateral margins widen slightly then curve inwards, posterior margin broadly truncate, without median point.

Antennula comprised of 7 articles; articles 1 and 2 distinct and articulated; article 2 0.8 times as long as article 1; article 3 as long as wide, 0.5 times as long as combined lengths of articles 1 and 2; last article terminating in 4–7 short simple setae. Antenna comprised of 7 articles; article 3 1.2 times as long as article 2, 2.1 times as long as wide; article 4 2.3 times as long as wide, 0.9 times as long as article 3; article 5 0.7 times as long as article 4, 1.7 times as long as wide; last article terminating in 6–7 short simple setae.

Molar process present, mandible palp without setae. Maxillula with 4 terminal robust setae. Maxilla lateral lobe with 2 recurved robust setae; mesial lobe with 2 large recurved robust setae. Maxilliped weakly segmented, palp article 2 with no simple setae, article 3 with 4 recurved robust setae and no simple setae.

Pereopod 1 basis 1.2 times as long as greatest width; ischium 0.9 times as long as basis; merus proximal margin with slight bulbous protrusion; carpus with straight proximal margin; propodus 1.3 times as long as wide; dactylus slender, 1.1 times as long as propodus, 2.3 times as long as basal width. Pereopod 7 basis 1.9 times as long as greatest width; ischium 0.9 as long as basis, without protrusions; merus proximal margin without bulbous protrusion, 0.5 as long as ischium, 0.9 times as long as wide; carpus 0.9 as long as ischium, without bulbous protrusion, 1.1 times as long as wide; propodus 0.8 as long as ischium, 1.7 times as long as wide; dactylus slender, 0.9 as long as propodus, 2.4 times as long as basal width. Pereopod 7 with small indentations on the inner side of the ischium, merus and carpus.

Pleopod 1 exopod as long as wide, lateral margin strongly convex, distally broadly rounded, mesial margin strongly convex; endopod 1.2 times as long as wide, lateral margin weakly convex, distally narrowly rounded, mesial margin straight, peduncle 0.7 times as wide as long. Pleopods 2–5 similar in structure to pleopod 1. Large medial lobes present and increasing in size from pleopods 1 to 5. Peduncle lobes increasing in size from pleopods 2 to 5.

Uropod longer than pleotelson; peduncle 0.7 times longer than exopod, lateral margin without setae; rami extending beyond pleotelson, marginal setae absent, apices broadly rounded. Endopod apically slightly pointed, 3.6 times as long as greatest width, lateral margin weakly convex, mesial margin weakly convex, terminating without setae. Exopod extending beyond endopod, 1.9 times longer than endopod, 3.8 times as long as greatest width, apically rounded, lateral margin straight, mesial margin straight, terminating without setae.

##### Type material.

Holotype (16.2 mm TL) from the gill cavity of *Tylosurus crocodilis crocodilis* from Bahia Mochima, Venezuela (USNM 216274); Paratypes (USNM 216275–216278) (Bruce 1986; not examined).

##### Distribution.

Off the coast of Florida, Florida Keys (USA); Cumaná, Venezuela (Bruce 1986, Bunkley-Williams et al. 1998, Schotte et al. 2009); and St. John Island, US Virgin Islands (present study).

##### Hosts.

Known from the hound needlefish, *Tylosurus crocodilis crocodilis* (Péron & Lesueur, 1821) (Bruce 1986, Bunkley-Williams et al. 1998, Schotte et al. 2009) and *Strongylura marina* (Walbaum, 1792) (previously unconfirmed but verified in the present study). There is also another unconfirmed record from *Strongylura notata notata* (Poey, 1860) in Florida (Bruce 1986).

##### Remarks.

*Mothocya xenobranchia* is known from Belonidae fish hosts and distinguished by the broad body which is arched in lateral view, the invaginations on the inner portion of pereopod 7, antenna with seven articles, and the shape of the pleotelson which is tapered anteriorly, then widens before bluntly rounding off.

When comparing *Mothocya xenobranchia* from the present study to the description given by Bruce (1986) there are a few minor differences but these are within the normal range of species variation. Variations include a different length to width ratio of the body and size of the eyes on the cephalon, more pronounced rostrum in the holotype, different number of setae on maxilla, but these characteristics given by Bruce (1986) are averages based on many specimens and can be variable depending on the specimen. In his remarks on the species, Bruce (1986) states the antenna can have seven or eight articles too and thus even this difference can be accounted for.

The other Caribbean species differ from *Mothocya xenobranchia* in that *Mothocya bermudensis* is smaller overall, with smaller eyes and less produced coxae; *Mothocya argenosa* has larger eyes, a larger and rounder pleotelson and smaller coxae; and *Mothocya nana* has a narrower body shape and is not arched longitudinally. *Mothocya bohlkeorum* has a narrow strongly produced rostrum; antennula and antenna bases closer together; larger and rounder coxae; and less developed proximomedial and peduncle lobes on the pleopods. Lastly, *Mothocya omidaptria* has much longer uropods, is not arched in lateral view, acute coxae on pereonite 7, and a narrowly produced rostrum. Furthermore, these species all have different hosts to *Mothocya xenobranchia* and thus there is no overlap of this isopod species on its host species in the Caribbean.

This record of *Mothocya xenobranchia* in the US Virgin Islands is a new locality record and also confirms the previously uncertain host record of *Strongylura marina* (Bruce 1986). The locality record conforms to the distribution of this species within the western Atlantic. Likewise, the host record is also from a Belonidae species and thus conforms to the host preference of this species.

**Figure 1.**
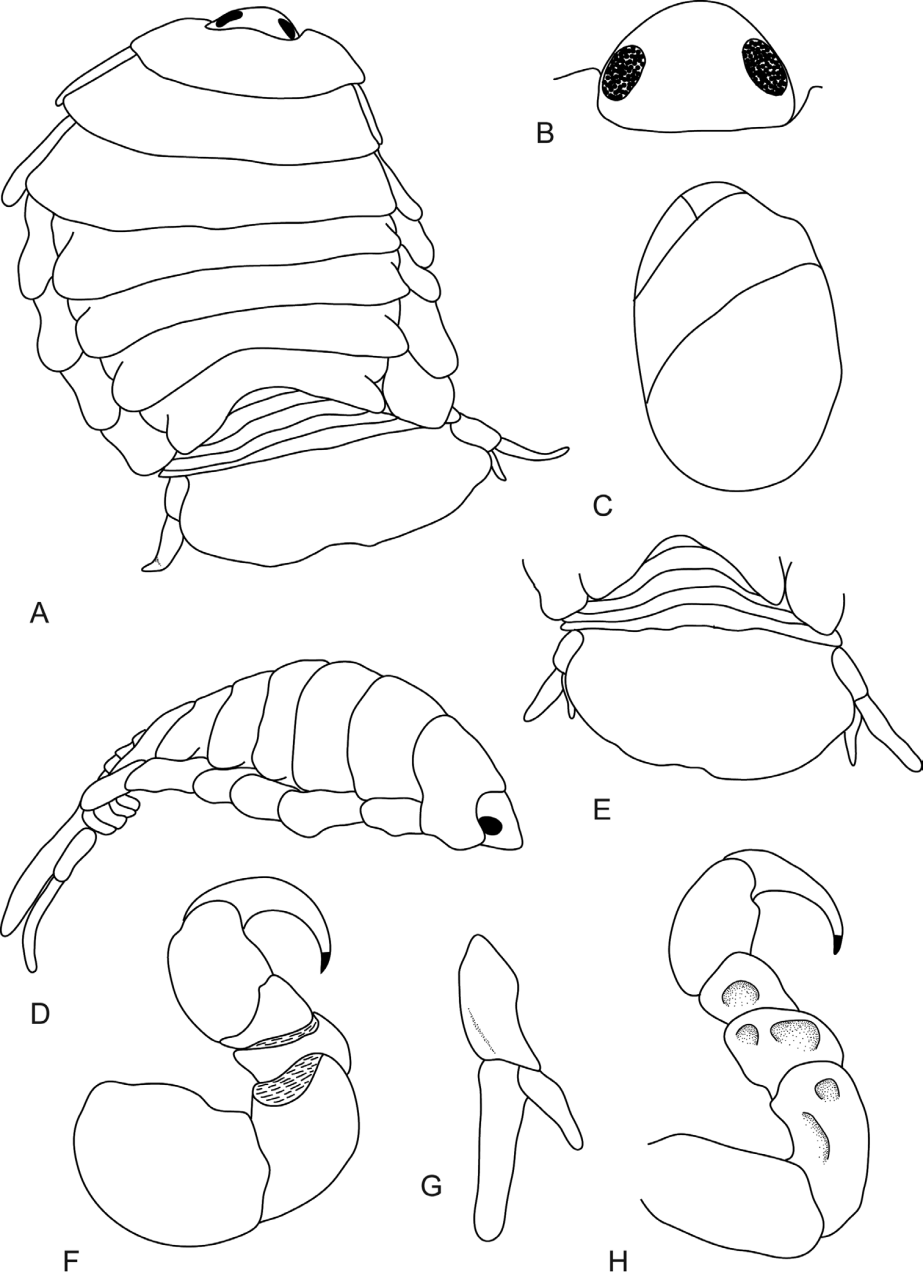
*Mothocya xenobranchia* Bruce, 1986 (15 mm) (AMNH_IZC 00197448): **A** dorsal view **B** dorsal view of cephalon **C** oostegites **D** lateral view **E** dorsal view of pleotelson **F** pereopod 1 **G** uropod **H** pereopod 7 showing indentations.

**Figure 2.**
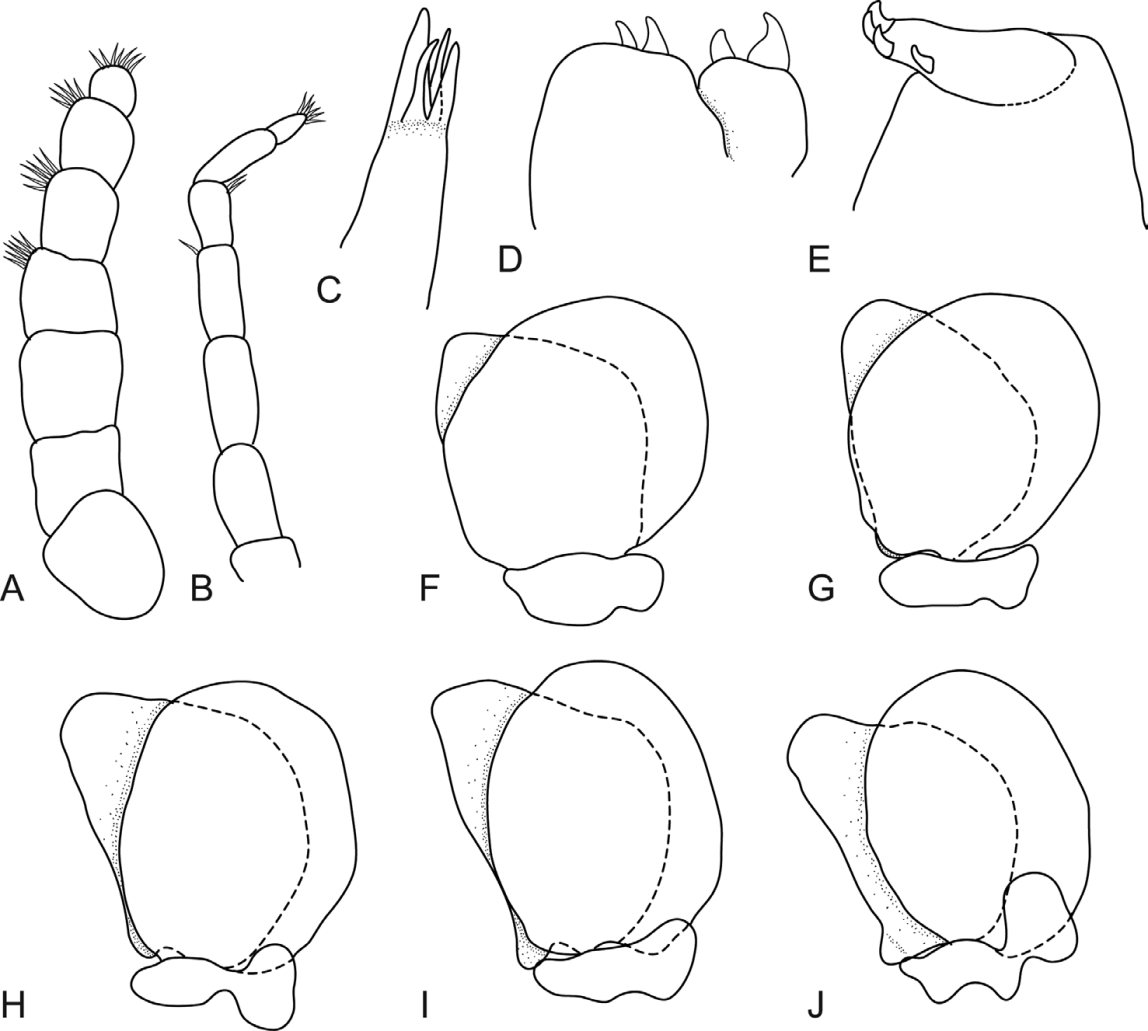
*Mothocya xenobranchia* Bruce, 1986 (15 mm) (AMNH_IZC 00197448): **A** antennula **B** antenna **C** tip of maxillula **D** tip of maxilla **E** tip of maxilliped article 3 **F** pleopod 1 **G** pleopod 2 **H** pleopod 3 **I** pleopod 4 **J** pleopod 5.

#### Mothocya bertlucy sp. n.

Taxon classificationAnimaliaIsopodaCymothoidae

http://zoobank.org/DC08E45E-5DDF-40D5-9310-B3AEA5C68265

**Figure 3.**
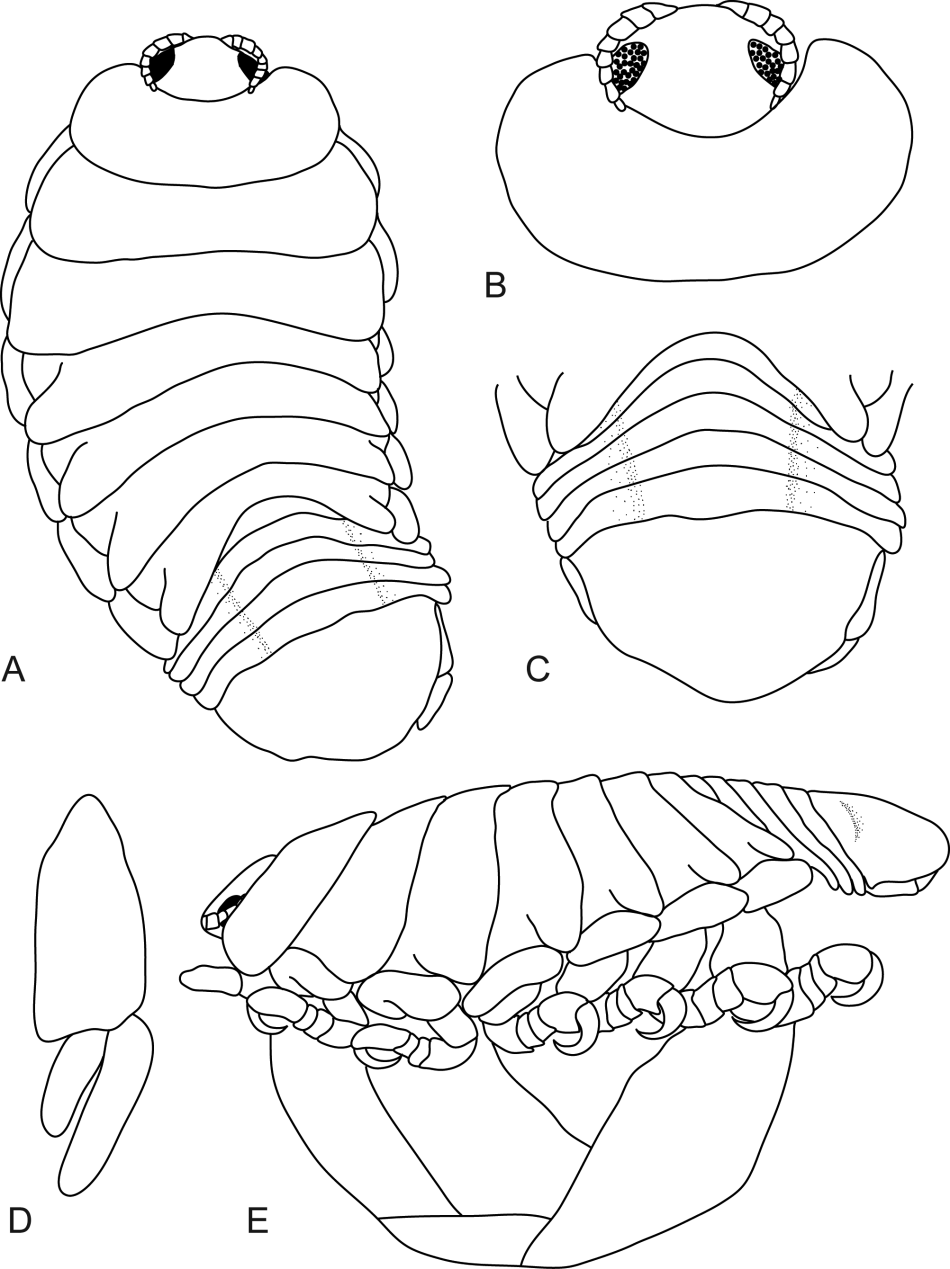
*Mothocya bertlucy* sp. n. ovigerous female holotype (7 mm) (AMNH_IZC 00197449): **A** dorsal view **B** anterodorsal view of pereonite 1 and cephalon **C** dorsal view of pleotelson **D** uropod **E** lateral view.

Figs 3 [Fig F4] [Fig F5] [Fig F6] –7

##### Material examined.

All material from the gills of the redlip blenny, *Ophioblennius macclurei*.

Holotype. Ovigerous ♀ (8.0 mm TL; 4.5 mm W), collected from Lameshur Bay, 18°18'59"N, 64°43'25"W, St. John Island, US Virgin Islands, July 2013, coll. L. Renoux & J. Sellers (AMNH_IZC 00197449).

Paratypes. ♀ dissected (7.0 mm TL; 3.5 mm W), three immature ♂♂, one dissected (5.5–6.0 mm TL; 2.0–2.5 mm W), collected from Brewers Bay, 18°20'24"N, 64°58'44"W, St. Thomas Island, Caribbean Sea), 19 May 2013, coll. J. A. Barry & A. McCammon (AMNH_IZC 00197450). Ovigerous ♀ (9.0 mm TL; 5.0 mm W), collected from Lameshur Bay, 18°18'59"N, 64°43'25"W, St. John Island, US Virgin Islands, July 2013, coll, L. Renoux & J. Sellers (AMNH_IZC 00197451). Ovigerous ♀ (7.5 mm TL; 4.0 mm W), mature ♂ (6.0 mm TL; 4.0 mm W), collected from Guana Island, 18°28'0"N, 64°33'59"W, British Virgin Islands, 07 July 2013, coll: R. Ditter & J. Barry (AMNH_IZC 00197452).

###### Ovigerous female holotype.

Body oval and moderately twisted, 1.9 times as long as greatest width, widest at pereonite 3, most narrow at pereonite 1, lateral margins slightly convex. Cephalon 0.7 times longer than wide, visible from dorsal view. Eyes oval with distinct margins, 0.2 times width of cephalon, 0.4 times length of cephalon. Pereonite 1 smooth, anterolateral angle rounded. Posterior margins of pereonites smooth and slightly curved laterally. Coxae narrow with rounded point, shorter or same length as pereonite. Pereonites 1–3 increasing in length and width; 4–7 decreasing in length and width, becoming progressively rounded posteriorly. Pleon with pleonite 1 largely concealed by pereonite 7, visible in dorsal view; pleonites posterior margin smooth, mostly concave; pleonite 2 partially overlapped by pereonite 7; pleonite 5 posterior margin slightly concave. Pleotelson 0.6 times as long as anterior width, dorsal surface smooth, lateral margins weakly concave, posterior margin converging to blunt caudomedial point.

Antennula comprised of 8 articles; articles 1 and 2 distinct and articulated with plumose setae; article 2 0.9 times as long as article 1; article 3 1.2 times as long as wide, 0.5 times as long as combined lengths of articles 1 and 2 with plumose seta; short simple setae present on last four articles, last article terminating in 4–8 short simple setae. Antenna comprised of 9 articles; article 3 1.3 times as long as article 2, 1.3 times as long as wide; article 4 1.4 times as long as wide, 1.1 times as long as article 3; article 5 as long as article 4, 1.4 times as long as wide; short simple setae on last three articles, last article terminating in 6–7 short simple setae.

Molar process present, mandible palp without setae. Maxillula with 4 terminal robust setae. Mxilla lateral lobe with 2 recurved robust setae; mesial lobe with 2 large recurved robust setae. Maxilliped comprised of 3 articles, palp article 2 without simple setae, article 3 with 3 recurved robust setae, and no simple setae.

Pereopods without robust or simple setae. Pereopod 1 basis 1.8 times as long as greatest width; ischium 0.6 times as long as basis; merus proximal margin without bulbous protrusion; carpus with straight proximal margin; propodus 1.4 times as long as wide; dactylus slender, 1.3 times as long as propodus, 2.6 times as long as basal width. Pereopod 2 propodus 1.3 as long as wide; dactylus 1.3 as long as propodus. Pereopod 7 basis 1.7 times as long as greatest width; ischium 0.7 as long as basis, without protrusions; merus proximal margin with slight bulbous protrusion, 0.4 as long as ischium, 0.6 times as long as wide; carpus 0.9 as long as ischium, without bulbous protrusion, 0.6 times as long as wide; propodus 0.9 as long as ischium, 1.3 times as long as wide; dactylus slender, 1.7 as long as propodus, 2.7 times as long as basal width.

Pleopod 1 exopod 1.3 times as long as wide, lateral margin weakly convex, distally narrowly rounded, medial margin weakly oblique, mesial margin strongly convex; endopod 1.8 times as long as wide, lateral margin weakly convex, distally narrowly rounded, mesial margin straight, peduncle 0.4 times as wide as long. Pleopods 2–5 similar to pleopod 1. Proximomedial lobes present and increasing in size from pleopod 1 to 5. Peduncle lobes absent.

Uropod more than half the length of pleotelson, peduncle 1.2 times longer than rami, peduncle lateral margin without setae; rami not extending beyond pleotelson, marginal setae absent, apices broadly rounded. Endopod apically rounded, 2.8 times as long as greatest width, lateral margin straight, mesial margin straight, terminating without setae. Exopod extending beyond endopod, 1.7 times longer than endopod, 4.2 times as long as greatest width, apically rounded, lateral margin straight, mesial margin straight, terminating without setae.

**Figure 4. F4:**
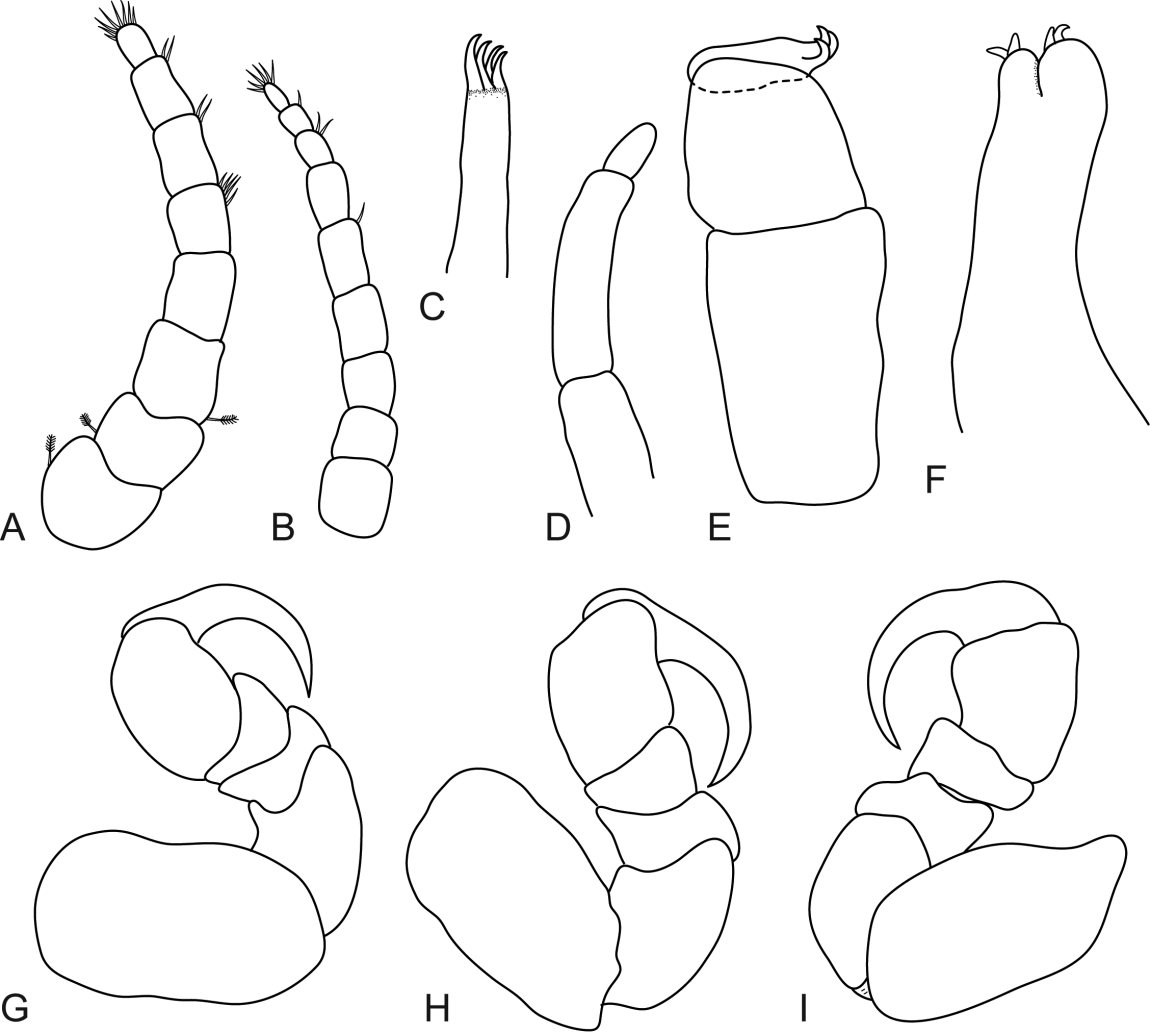
*Mothocya bertlucy* sp. n. female paratype (7 mm) (AMNH_IZC 00197450): **A** antennula **B** antenna **C** maxillula **D** molar process **E** maxilliped **F** maxilla **G** pereopod 1 **H** pereopod 2 **I** pereopod 7.

**Figure 5. F5:**
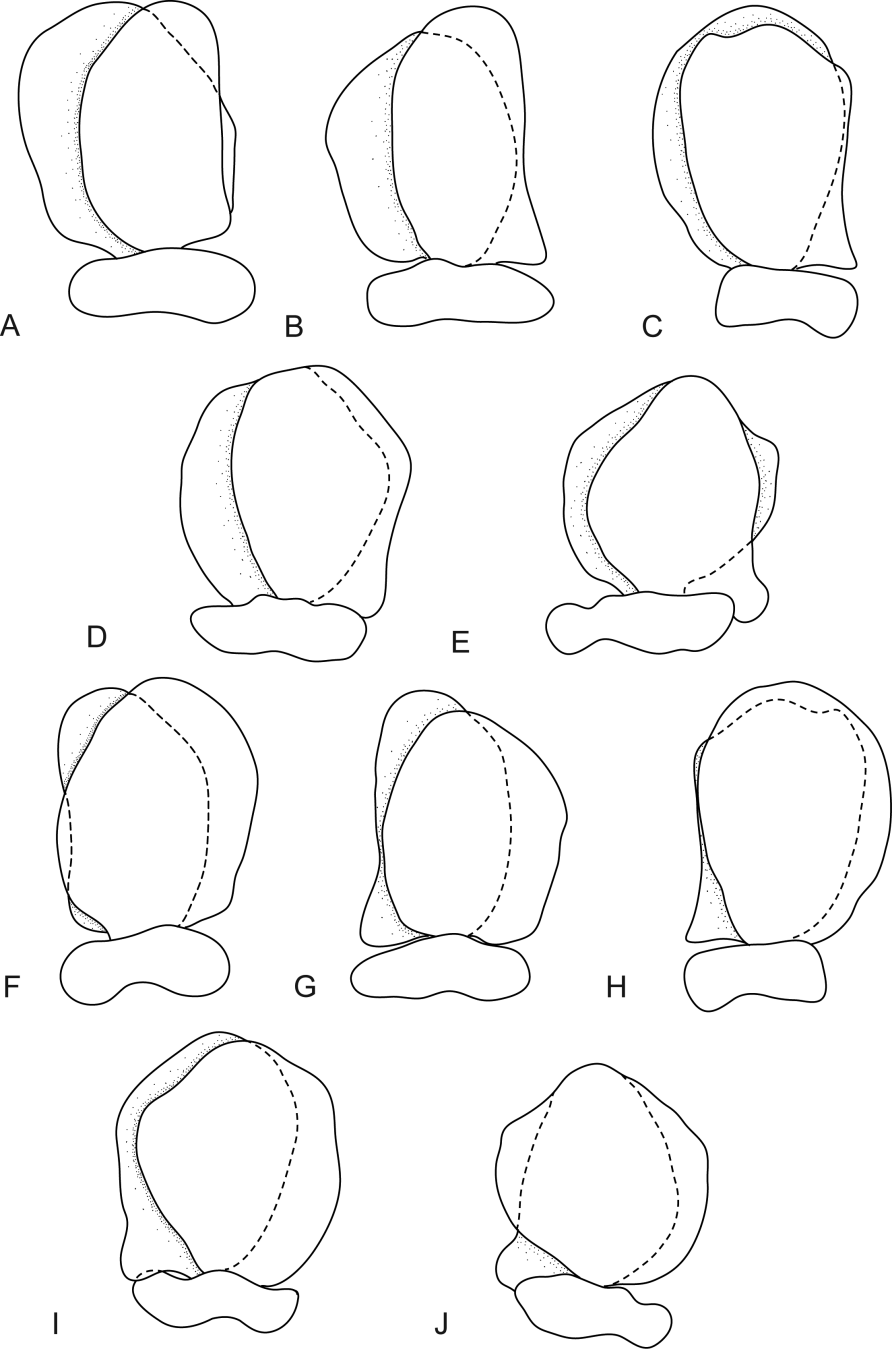
*Mothocya bertlucy* sp. n. female paratype (7 mm) (AMNH_IZC 00197450): **A–E** dorsal pleopod 1–5 respectively **F–J** ventral pleopod 1–5 respectively.

###### Male.

Males similar to females but smaller. Body more oval and not twisted, 2.1 times as long as wide. Maxilliped article three with three recurved robust setae. Maxilla with one recurved robust seta on the medial lobe and two on the lateral lobe. Penis set close together, medially united. Pleopod 2 appendix masculina basally swollen, 0.8 times as long as endopod, distally bluntly rounded. Pleotelson triangular converging to a sharp caudal point. Uropods extend past posterior margin of pleotelson and endopod is longer, exopod 1.5 times as long as endopod.

**Figure 6. F6:**
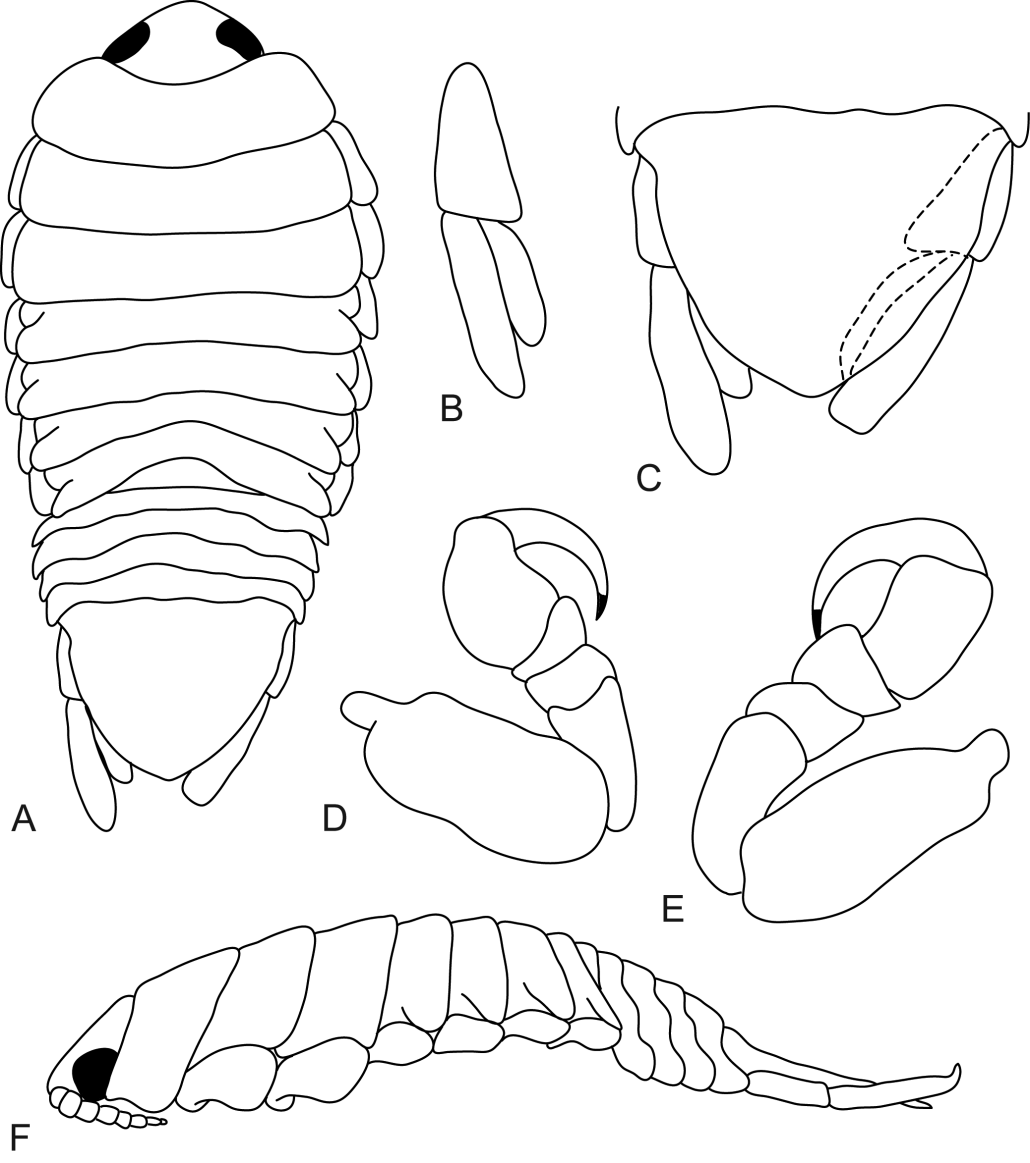
*Mothocya bertlucy* sp. n. male paratype (5.5 mm) (AMNH_IZC 00197450): **A** dorsal view **B** uropod **C** dorsal view of pleotelson **D** pereopod 1 **E** pereopod 7 **F** lateral view.

**Figure 7.**
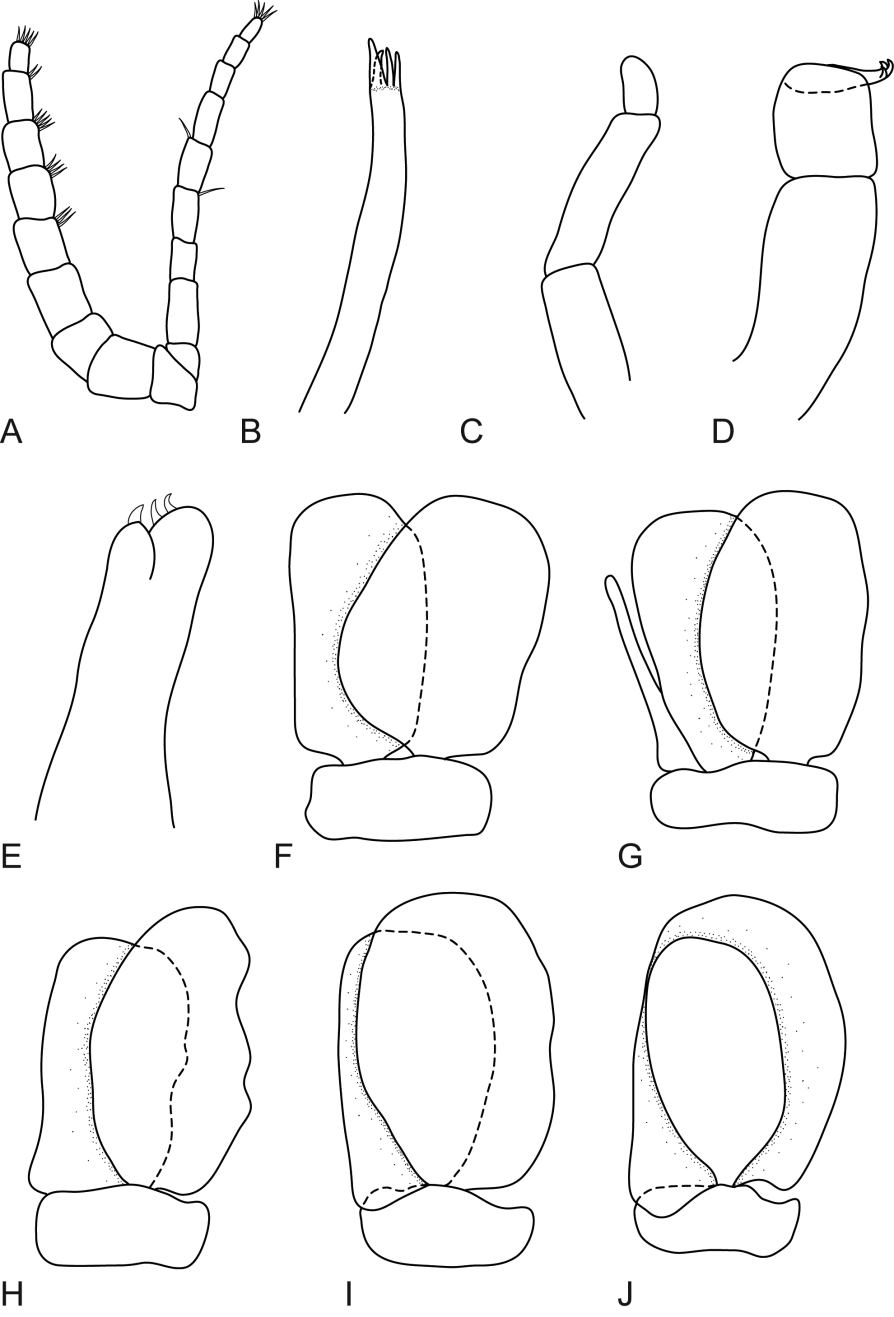
*Mothocya bertlucy* sp. n. male paratype (5.5 mm) (AMNH_IZC 00197450): **A** antennula and antenna **B** maxillula **C** molar process **D** maxilliped **E** maxilla **F–J** pleopod 1–5 respectively.

##### Size.

Ovigerous females (7.0–9.0 mm TL; 3.5–5.0 mm W), non-ovigerous females (7.0 mm TL; 3.0 mm W); mature male (6.0 mm TL; 4.0 mm W), immature males (5.5–6.0 mm TL; 2.0–2.5 mm W).

##### Etymology.

This species is named in honour of Ernest H. (“Bert”) Williams Jr. and Lucy Bunkley-Williams on the occasion of their retirement and in recognition of their contribution to Caribbean marine parasitology; noun in apposition.

##### Distribution.

Known from St. John, St. Thomas, and Guana Islands, Caribbean Sea.

##### Hosts.

Only known from the redlip blenny, *Ophioblennius macclurei* (Silvester, 1915).

##### Remarks.

*Mothocya bertlucy* sp. n. can be identified by its unique host (redlip blenny), small size (like those reported from atherinids), relatively small eyes, the small pleotelson with a narrowly rounded caudomedial point, large uropod peduncle with short rami, uropods which do not extend past the pleotelson posterior margin, and the narrow pleon which is only slightly overlapped by pereonite 7.

The species most similar to *Mothocya bertlucy* sp. n. is *Mothocya rosea* Bruce, 1986 found on the Mexican and Californian coasts. In comparison to *Mothocya bertlucy*, *Mothocya rosea* has more produced proximomedial lobes on pleopods 3–5, larger eyes, broad truncate pleotelson, and four setae on the maxilliped article 3.

The three small *Mothocya* species from atherinids (*Mothocya argenosa*; *Mothocya epimerica*; and *Mothocya waminda* Bruce, 1986) were all compared to the current species. *Mothocya argenosa* from the western Atlantic measures 5.6–9.8 mm, but has larger eyes, longer uropods, the pleotelson is more rounded and the posterolateral margins of pereonite 7 are acute. *Mothocya epimerica* from the Mediterranean has a more pointed rostrum, rounded pleotelson, larger eyes and four setae on the maxilliped. *Mothocya waminda* from the Indo-Pacific has an appendix masculina on pereopod 2 in the female and longer uropods.

*Mothocya bertlucy* sp. n. differs from all the other known Caribbean species in that *Mothocya bohlkeorum* has much larger and more produced coxae and a larger truncate pleotelson; *Mothocya nana* has a wider pleotelson, truncate rostrum and larger coxae; *Mothocya bermudensis* has an antennula with only seven articles, large eyes and an arched body; and *Mothocya omidaptria* has longer uropods extending past the pleotelson, a strongly produced rostrum and acute coxae as well as posterolateral angles of pereonite 7.

This is the first account of a *Mothocya* species from the US Virgin Islands and is also the first record on a blenny, which helps establish its status as a new species as Bruce (1986) commented that “host identity may be useful in making a *Mothocya* identification.”

## Supplementary Material

XML Treatment for Mothocya

XML Treatment for Mothocya xenobranchia

XML Treatment for Mothocya bertlucy
